# Optimisation modelling to improve the diets of First Nations individuals

**DOI:** 10.1017/jns.2019.30

**Published:** 2019-09-20

**Authors:** Louise Johnson-Down, Noreen Willows, Tiff-Annie Kenny, Amy Ing, Karen Fediuk, Tonio Sadik, Hing Man Chan, Malek Batal

**Affiliations:** 1 Département de nutrition, Faculté de Médecine, Université de Montréal, Pavillon Liliane de Stewart, CP 6128 succ. Centre-Ville, Montréal, QC, Canada H3T 1A8; 2 Department of Agricultural, Food and Nutritional Science, University of Alberta, 410 Agriculture/Forestry Centre, Edmonton, AB, Canada T6G 2P5; 3 Department of Biology, University of Ottawa, 30 Marie Curie, Ottawa, ON, Canada K1N 6N5; 4 First Nations Food, Nutrition and Environment Study, University of Ottawa, Ottawa, ON, Canada K1N 6N5; 5 Assembly of First Nations, Ottawa, ON, Canada K1P 6L5; 6 WHO Collaborating Centre on Nutrition Changes and Development, Université de Montréal, Pavillon Liliane de Stewart, CP 6128 succ. Centre-Ville, Montréal, QC, Canada H3T 1A8

**Keywords:** First Nations, Indigenous peoples, Diet optimisation, Diet modelling, Linear programming, Nutrition transition, CNF, Canadian Nutrient File, EAR, estimated average requirements, FNFNES, First Nations Food, Nutrition and Environment Study, MARKFOOD, healthy market foods, M.F., milk fat, TRADFOOD, nutrient-dense traditional food, UL, tolerable upper intake level

## Abstract

We examined the feasibility of linear programming (LP) to develop diets that were economical, included traditional (cultural, non-market) foods and met the dietary reference intakes (DRI) in a Canadian Indigenous population. Diet optimisation using LP is a mathematical technique that can develop food-based dietary guidelines for healthy eating in Indigenous populations where food insecurity, availability and cost are important considerations. It is a means of developing nutritionally optimal food combinations that are based on economical and culture-specific foods. Observed food consumption data were derived using 24-h food recalls from the First Nations Food, Nutrition and Environment Study. The LP models were constructed to develop diets meeting DRI, cost and food constraints. Achieving the recommended food intake was not feasible in a model meeting all nutrient requirements. Models that met most nutrient requirements at reduced cost were designed for men and women, separately. In women, it was necessary to increase energy intake to meet most nutrient requirements. Nutrient requirements could not be met for fibre, linoleic and linolenic acids, vitamin D, Ca and K in both sexes, P in women, and Mg and vitamin A in men. Using LP to develop optimal diets for First Nations people, we found simultaneous achievement of all DRI was difficult, suggesting that supplementation might be necessary which goes against recommendations for individuals to meet their nutrient needs through healthy eating patterns. Additionally, to make diets feasible, programmes to reduce market food costs and to support First Nations people in traditional food harvesting are recommended.

Globally, Indigenous Peoples, including First Nations in Canada, are more likely to experience poverty, health challenges, obesity and nutrition-related chronic disease (e.g. type 2 diabetes) than their non-Indigenous counterparts^(1–6)^. First Nations are one of the three Indigenous groups recognised in the Canadian constitution^(7)^. The prevalence of type 2 diabetes is three to five times higher in First Nations than among other Canadians (age-standardised prevalence diabetes rate of 17·2 % in on-reserve First Nations *v.* 5 % in the non-Indigenous population^(6)^). The personal burden as well as the health care costs associated with type 2 diabetes will worsen since population growth among First Nations is four times that of the general Canadian population^(8)^.

The high prevalence of nutrition-related chronic disease in First Nations can mostly be explained by the high prevalence of food insecurity that they experience, meaning that they have insufficient physical and financial access to both sufficient healthy market foods (MARKFOOD) as well as culturally appropriate, nutrient-dense traditional food (TRADFOOD) procured from local ecosystems, such as game, fish, fowl and plants^(8,9)^. There are several explanations for the limited access to TRADFOOD and high-quality MARKFOOD such as fruits, vegetables and unprocessed meat. One reason is that European colonial policies and practices undermined or destroyed land-based food practices and created barriers for Indigenous communities to access sufficient healthy foods from local food systems^(10)^. Another reason is that many First Nations have lower incomes than other Canadians and therefore less money available to purchase nutrient-dense high-quality MARKFOOD^(11)^. Unprocessed nutrient-dense foods like vegetables tend to cost more per g and per kJ than nutrient-poor processed food^(12)^. Furthermore, the overall cost of obtaining store-bought or MARKFOOD is high in First Nations reserve communities located in rural or remote areas and the quality of fresh foods is poor as a result of spoilage and transportation challenges^(13)^.

Considering the health, financial and cultural challenges facing First Nations peoples, it is imperative to provide culturally appropriate, practical recommendations to improve their diet. Improving diet could lessen health concerns in this population at high risk for obesity, CVD and diabetes^(1,14,15)^. Linear programming is a mathematical technique that can be used to convert nutrient, cost and food constraints into food-based dietary guidelines for healthy eating in populations where food security and cost are important considerations. It can generate optimal solutions that satisfy several constraints including meeting nutrient requirements at low cost and conforming to typical eating habits in the target population including important TRADFOOD (i.e. remain as close to the population diet as possible)^(16,17)^. The advantage of this technique is that the existing diet of a population is the basis for modelling; thus, optimal diets contain familiar foods. Because this technique can integrate many variables and constraints, it is well suited to the complex task of developing dietary guidelines in challenging environments such as those that exist in many First Nations communities^(17)^.

Our objective was to examine the possibility of meeting Institute of Medicine nutrient requirements for macro- and micronutrients in healthy persons^(18–23)^ based on the foods available and consumed in First Nations reserve communities in Ontario, at no additional cost (i.e. the cost of the theoretical diet must be equal to, or below, the estimated cost of the observed diet). In the province of Ontario, 47·6 % of First Nations adults living on reserves report living in food-insecure households (marginal, moderate and severe household food insecurity, combined)^(24)^. Although this approach to diet modelling has been used previously to formulate cost-efficient and favourable dietary patterns in other populations^(16,25,26)^, to our knowledge, it has never been used to optimise diets in First Nations individuals in Canada.

## Methods

### Study participants

Data were collected in the autumn of 2011 and 2012 from participants in eighteen reserve communities in the province of Ontario, as part of the First Nations Food, Nutrition and Environment Study (FNFNES)^(27)^. The details of FNFNES have been described elsewhere^(27–29)^. Briefly, FNFNES was a pan-Canadian study that aimed to address gaps in knowledge regarding diet and environment among First Nations adults living on-reserve south of the 60th parallel in Canada. FNFNES data represent the most complete dietary information available from a representative sample of First Nations individuals to ever be collected in Ontario. Informed consent was obtained from all participants. This study was conducted according to the guidelines laid down in the Declaration of Helsinki and all procedures involving human participants were approved by the research ethics boards of Health Canada, University of Northern British Columbia, University of Ottawa and Université de Montréal. Written informed consent was obtained from all participants.

Of the eighteen communities included in the analyses, seven (39 %) were remote (fly-in or winter road only), two (11 %) were rural or ≥60 km away from the nearest urban centre and nine were within 60 km of an urban centre. Of the 1919 households of the participating First Nations communities in Ontario, thirty-one did not have eligible participants (First Nations, 19 years or older, or living on-reserve; without health conditions such as deafness or cognitive impairment) and seventy-nine were vacant. Of the remaining 1809 households, 380 refused to participate or had incomplete interviews. There were 1429 complete interviews; however, after excluding pregnant and breast-feeding women (*n* 40) and individuals with no food intake the prior day (*n* 2), the final sample size for the present study was 1387 interviews.

### Household food insecurity

A food security questionnaire adapted from the Household Food Security Module used in the USA^(30)^ for use in Canada and Indigenous individuals was conducted^(31)^. Households were classified as food secure when they gave no indication of income-related food access. Classifications of marginal (some concern or problem with food access), moderate (compromises in the quality and/or quantity of foods) and severe (reduced food intake due to lack of money) food insecurity were included in estimates^(32)^.

### Dietary data

For each participant, dietary intake was assessed using a single 24-h recall. Dietary recalls were conducted by trained community interviewers using a three-stage multiple-pass method, i.e. quick list, detailed description and review^(33)^. Three-dimensional food models were used to estimate portion sizes (Santé Québec, Montréal, Québec, Canada). Dietary recalls were entered by research nutritionists at the Université de Montréal using CANDAT (Godin, London, Ontario, Canada), a nutrient analysis software utilising the 2010 Canadian Nutrient File (CNF)^(34)^.

The accuracy of 24-h recall data entry was ensured by cross-checking 10 % of the records and conducting preliminary analyses to detect outlying food and nutrient intakes (defined as ±2 sd of the mean for energy and nutrients)^(29)^. All errors were reconciled before data analyses.

All missing nutrients from the 2010 CNF were imputed using the 2015 CNF^(35)^ and the United States Department of Agriculture Food and Nutrient Database for Dietary Studies^(36)^. Nutritional information about TRADFOOD and MARKFOOD that was not available from the CNF was manually added to the database by an FNFNES analyst^(37)^.

### Food groupings

Diet modelling requires the categorisation of foods into food groupings for input into the program. A total of 1705 foods consumed by the participants were classified into forty-nine food groups (Table 1). Dairy food groups were categorised into high-fat and low-fat versions (low-fat fluid milk, yogurt with ≤2 % milk fat (M.F.) and low-fat cheese with ≤20 % M.F.)^(38)^. Since Hg contamination is a concern in certain First Nations communities traditional fish from local lakes and rivers was classified into high and low Hg content based on a cut-off of 0·5 µg/g^(39)^. The mean daily portion size (g/person per d) from the observed diet for each of the forty-nine food groups was obtained from the recalls. The composite nutrient profile of each of the food groups was calculated by weighting the proportional consumption of the foods contained in each food group by the weight (g) of food for thirty nutrients (see list in Table 2). The rationale for this approach is that a food eaten more often would have a greater impact on the nutrient intake^(40–42)^. Diet beverages (e.g. soda) were included but not other non-energy foods such as coffee, tea and water.

**Table 1.** Food groups used for modelling intake and proportion of 1387 First Nations individuals from Ontario, Canada consuming each food group

		Women		Men		Total population
Food category*	Foods included	g/d	%	g/d	%	%
Diet drinks	Diet drinks sweetened with non-energy sweeteners	76·9	13·6	61·6	10·2	12·3
Sweet drinks	All sweetened drinks including soft drinks, fruit punches, etc.	265·8	42·2	345	43·9	42·8
Coffee whitener	Non-dairy coffee creamers, powder and liquid	1·44	16·9	2·60	21·7	18·7
High-fat cheese	Higher fat (>20 % M.F.) cheese including cream and processed cheese	8·41	25·8	9·06	19·4	23·4
Low-fat cheese	Low-fat (≤20 % M.F.) cheese including cream and processed cheese	1·27	1·40	0·09	0·38	1·01
Cream	Cream including whipped	9·60	21·6	10·4	19·4	20·8
Evaporated milk	Canned evaporated milk including sweetened	4·79	11·3	6·05	15·4	12·9
High-fat fluid milk	Higher-fat (>2 % M.F.) fluid and dry milk including chocolate but excluding evaporated	6·45	3·03	7·07	2·25	2·74
Low-fat fluid milk	Low-fat (≤2 % M.F.) fluid and dry milk including chocolate but excluding evaporated	63·9	39·5	75·3	36·6	38·4
High-fat yogurt	Higher-fat (>2 % M.F.) yogurt and soured cream	5·29	4·67	0·95	1·70	3·53
Low-fat yoghurt	Low-fat (≤2 % M.F.) yogurt and soured cream	8·49	4·79	4·91	2·64	3·97
Salad dressing	Mayonnaise, mayonnaise-type spreads, tartar sauce, salad dressings including vinegar	3·80	20·0	5·24	16·4	18·6
Shortening and oil	Shortening, lard and vegetable oils	3·15	20·3	3·98	24·7	22·0
Margarine and butter	Butter and margarine	8·20	49·5	12·5	55·0	51·6
Fruit juice	100 % Fruit juices	39·0	11·2	37·4	8·29	10·1
Fruit	Fresh, frozen, canned and dried fruits (not including berries that would be gathered in Indigenous communities)	67·8	28·4	38·3	20·2	25·2
Bannock	Bannock	9·90	6·78	15·5	6·59	6·71
High-fibre hot cereal	High-fibre hot cereals such as oatmeal and cream of wheat† (>4 g in 100 g)	0·70	0·70	0·98	0·94	0·79
Low-fibre hot cereal	Low-fibre hot cereals such as oatmeal and cream of wheat† (≤4 g in 100 g)	50·2	13·4	61·1	14·5	13·8
High-fibre cereal ready to eat	High-fibre ready to eat cereals (>4 g in 100 g)	6·42	9·93	6·75	8·29	9·30
Low-fibre cereal ready to eat	Low-fibre ready-to-eat cereals (≤4 g in 100 g)	2·61	5·37	2·44	4·33	4·97
Pasta/rice	Plain pasta or rice	46·2	29·8	69·2	30·9	30·2
Potatoes	All potatoes including steamed, boiled, baked, French fried, mashed and hash browns	58·7	45·3	88·1	46·0	45·6
Whole-grain bread	Whole-grain breads, flour and leavening agents	17·9	28·9	26·7	31·1	29·7
White bread	White breads, flour and leavening agents	40·0	54·0	46·8	52·5	53·4
Mixed dishes	Combination dishes of meat or alternatives, grains, dairy products and vegetables such as lasagne, pizza, shepherd's pie, etc.	120·2	40·3	156	38·6	39·7
Meat alternatives	Legumes (beans), nuts, seeds and nut butters	10·4	16·8	12·6	10·5	14·4
Beef	Beef and veal (including jerky)	37·2	27·6	57·6	27·9	27·7
Eggs	Fried, boiled, poached and scrambled eggs	33·9	33·3	51·2	37·1	34·8
Fish, not canned	Fish and seafood (shrimp, crab, etc.) from the store including fresh, frozen	0·06	0·12	1·59	0·38	0·22
Fish, canned	Fish and seafood from the store, canned	1·78	2·92	1·44	1·70	2·45
Pork, not including ham	Pork not including ham	11·1	12·7	16·1	15·6	13·8
Poultry	Chicken and turkey including flour and batter fried	44·0	29·1	44·7	24·9	27·5
Processed meat	Processed meats such as hot dogs, bologna, salami, ham, sausages, etc.	27·6	37·6	38·7	44·4	40·2
Table salt and spices	Table salt, herbs, spices and flavoured toppings including soya sauce, dry and canned gravies	6·14	17·9	7·00	15·3	16·9
Table sauces	Ketchup, chili sauce, sweet and sour sauce, teriyaki sauce, etc.	4·56	15·3	5·12	17·1	16·0
Snacks	Chips, Cheetos, popcorn, crackers, etc.	12·8	29·2	15·9	24·9	27·5
Soup	Dry, canned and homemade soups	85·7	22·5	121	27·7	24·5
Cake, cookies and pie	Cakes, cookies, pies and granola bars	24·5	24·3	19·0	18·4	22·1
Candies	Candies such as chocolate bars, chewy and hard candy	9·09	10·5	6·38	6·00	8·80
Dairy desserts	Desserts made from milk products such as ice cream, dessert toppings, etc.	6·89	3·86	7·55	3·20	3·60
Sugar, jams and syrups	Granulated, brown and icing sugar, jams and syrups	12·7	51·3	22·4	59·7	54·5
Non-energy sweeteners	Non-energy sweeteners such as aspartame, etc.	0·50	17·1	0·47	13·4	15·6
High-Hg traditional fish	High-Hg (>0·5 µg/g) fish caught from rivers, lakes and bays in traditional territories	2·16	1·05	9·65	4·33	2·31
Low-Hg traditional fish	Low-Hg (≤0·5 µg/g) fish caught from rivers, lakes and bays in traditional territories	3·41	1·64	6·32	1·70	1·66
Traditional game meat	All traditional meats (i.e. hunted) of animal origin, i.e. large and small mammals, birds, as well as their organs	14·2	7·36	29·2	10·7	8·65
Traditional plants and fruit	Berries, traditional grains and wild plants	0·86	0·93	1·74	0·75	0·87
Vegetables, canned	Canned vegetables including vegetable juices	23·3	15·5	29·9	13·4	14·7
Vegetables, fresh	Fresh, dried and frozen vegetables	54·4	48·2	52·7	40·7	45·3

M.F., milk fat.

* Food categories used to define constraints of serving sizes in linear programming optimisation.

† The same hot cereals appear in both the high fibre and low fibre groups because the dry cereals meet the high fibre criterion of greater than 4 g per 100 g whereas the prepared cereals do not.

**Table 2.** Comparison of nutrient intake for observed and optimised diets in 1387 First Nations individuals from Ontario, Canada

	Women							Men						
Nutrients	Nutrient constraint†	Observed‡	% Constraint	Model 1§	% Constraint	Model 2‖	% Constraint	Nutrient constraint†	Observed‡	% Constraint	Model 1§	% Constraint	Model 2¶	% Constraint
Energy														
kJ	NA	7360	NA	8104	NA	7326	NA	NA	9422	NA	8368	NA	8205	NA
kcal	NA	1759	NA	1937	NA	1751	NA	NA	2252	NA	2000	NA	1961	NA
Carbohydrates (g)	100	207	207	197	197	226	226	100	255	255	293	293	255	255
Total sugar (g)	109	91·7	84·1	97·6	89·6	71·1	65·3	142	110	77·7	89·5	63·1	79·3	55·9
Free sugar (g)	43·8	63·1	144*	15·4	35·3	38·4	87·8	56·7	78·6	139	20·3	35·8	46·1	81·3
Total dietary fibre (g)	21·7	13·2	60·9*	21·7	100	18·3	84·3*	31·4	15·8	50·4*	31·4	100	19·7	62·7*
Fat (g)	68·1	72·5	107*	63·8	93·7	64·9	95·3	75·6	94·3	125	64·7	85·6	68·9	91·1
MUFA (g)	NA	28·3	NA	21·3	NA	24·7	NA	NA	37·1	NA	23·1	NA	26·1	NA
PUFA (g)	NA	14·7	NA	17·6	NA	15·1	NA	NA	19·0	NA	17·1	NA	16·0	NA
SFA (g)	19·4	23·2	120*	16·9	87·4	19·4	100	25·2	30·0	119	18·2	72·2	20·7	82·2
Linoleic acid (g)	11·7	12·6	108	11·7	100	13·0	111	14·5	16·2	112	14·5	100	13·7	94·6*
Linolenic acid (g)	1·1	1·58	144	1·59	145	1·55	NA	1·60	2·10	131	1·60	100	1·64	103
Cholesterol (mg)	NA	298	NA	302	NA	254	NA	0·00	422	NA	143	NA	272	NA
Protein (g)	37·6	75·0	199	153	407	72·9	194	46·2	102	221	81·1	175	89·4	193
Vitamin A (μg)	500	536	107	1361	272	502	100	625	662	106	1020	163	653	104
Thiamine (mg)	0·88	2·64	299	5·01	569	2·65	301	1·00	3·29	329	5·04	504	3·28	328
Riboflavin (mg)	0·88	1·54	175	2·60	296	1·48	169	1·10	1·98	180	1·85	168	1·69	154
Niacin (mg)	11·0	34·0	309	59·9	545	34·0	309	12·0	45·4	378	38·4	320	40·1	335
Vitamin B_6_ (mg)	1·21	1·43	118	2·7	220	1·61	133	1·10	1·81	164	2·77	252	1·91	174
Dietary folate equivalents (μg))	320	342	107	362	113	393	123	320	428	134	415	130	438	137
Vitamin B_12_ (μg)	1·98	5·12	258	14·0	707	4·48	226	2·00	7·58	379	6·30	315	7·41	370
Vitamin C (mg)	60	72·1	120	130	217	64·0	107	75·0	79·1	105	162	216	75·0	100
Vitamin D (μg)	10	4·23	42·3*	48·7	487	4·03	40·3*	10·0	6·19	61·9*	10·0	100	4·85	48·5*
Ca (mg)	878	548	62·4*	1363	155	522	59·5*	817	656	80·4*	817	100	553	67·7*
Cu (μg)	0·88	1·11	126	1·83	208	1·30	148	0·70	1·45	207	2·00	286	1·52	218
Fe (mg)	6·93	12·6	181	13·4	193	13·3	191	6·00	16·4	274	15·0	251	16·4	273
Mg (mg)	263	212	80·5*	425	162	263	100	347	267	77·0*	407	117	300	86·7*
P (mg)	2580	1082	41·9*	2580	100	1111	43·1*	580	1402	242	1488	257	1300	224
K (mg)	4700	2004	42·6*	4700	100	2295	48·8*	4700	2549	54·2*	4700	100	2852	60·7*
Na (mg)	1413	2750	195*	2300	163	2300	163	1410	3540	251*	2300	163	2300	163
Zn (mg)	6·82	10·5	154	11·9	174	9·81	144	11·00	14·5	132	11·2	102	13·4	122

NA, not applicable; AMDR, acceptable macronutrient distribution range; EAR, estimated average requirement; AI, adequate intake.

* Values are less than 100 % observed where there is a minimum and greater than 100 % where there is a maximum.

† Nutrient constraints used Institute of Medicine AMDR, EAR, AI or accepted guidelines.

‡ Observed is the actual observed diet of the participants.

§ Model 1 for both women and men was optimised using only nutrient constraints such as meeting EAR, AI, AMDR except maximum 30 % energy as fat, 10 % energy as free sugars, 25 % as energy of total sugars and 10 % energy as SFA.

‖ Model 2 for women included most nutrient constraints from model 1 except for Ca, fibre, P and K and additional constraints on foods limiting minimums to no less than half and maximums to double for most foods except diet and sweet drinks, coffee whitener, fats, high-sugar foods and processed meats that were limited to a maximum of the same amount as the observed.

¶ Model 2 for men included most nutrient constraints from model 1 with the exception of Ca, fibre, Mg, K and vitamins A and D and additional constraints as for the women.

### Food cost

The cost of foods was determined in two different ways. During the initial data collection in 2011–2012, the cost of sixty-seven basic food items contained in Health Canada's 2008 National Nutritious Food Basket Tool (Appendix 1)^(43)^ were collected and averaged across food retailers in the sampled communities. These costs were increased by 9·08 % to account for the cost of living increase^(44)^. Because the food basket did not include many pre-packaged meals consumed by First Nations (such as pizza, canned stew, etc.), spices and condiments, these costs were obtained from the Independent Grocers Alliance online grocery shopping website^(45)^ using a store location in Ottawa, Ontario, Canada recording only regular prices from 2018. To account for the higher costs in remote communities, we compared the cost of items in the Health Canada's 2008 National Nutritious Food Basket in the sampled communities with those in Ottawa and calculated an average price increment of 20·4 % that was applied to the costs obtained from the Independent Grocers Alliance website. Food cost for each of the forty-nine food groups created for this study was then calculated for the gram weight of the edible yield of the food and then weighted by the contribution to the total gram weights of all the foods in a food group^(25,41)^.

### Diet modelling

Optimal diet models were formulated to identify the dietary changes necessary for First Nations adults to achieve dietary reference intakes recommended by the Institute of Medicine^(18–23)^. Separate models were run for men and women because their dietary patterns and nutritional requirements are different. The models were designed to meet nutrient requirements while remaining as close to the mean observed population diet as possible (as reported in the 24-h recalls of the First Nations) (Table 3). This assumes that a population would be more likely to adopt a recommended diet if the foods were like the observed population diets and did not cost more^(46)^.

**Table 3.** Summary of optimisation model scenarios for 1387 First Nations individuals from Ontario, Canada*

	Model constraints			Model results	
Model	Nutrients†	Market foods‡	Traditional foods	Feasibility	Cost (Canadian $/d)
Men					
Model 1	Meet all nutrient requirements	Minimum observed intake ≤ grams of market food groups ≤90th percentile of observed intake	None	Feasible	9·54
Model, no limit to fish	Meet all nutrient requirements	½ observed intake ≤ grams of market food groups ≤2× observed intake	High-Hg fish ≤1× observed intake§	Infeasible	–
Model 2	Drop fibre, Ca, Mg, K, linoleic and linolenic acids, and vitamins A and D	½ observed intake ≤ grams of market food groups ≤2× observed intake	High-Hg fish ≤1× observed intake§	Feasible	8·63
Women					
Model, energy = observed	Meet all nutrient requirements	Minimum observed intake ≤ grams of market food groups ≤ 90th percentile of observed intake	None	Infeasible	–
Model 1	Meet nutrient requirements (relaxing energy)	Minimum observed intake ≤ grams of market food groups ≤90th percentile of observed intake	None	Feasible	13·47
Model, no limit to fish	Meet nutrient requirements (relaxing energy)	½ observed intake ≤ grams of market food groups ≤2× observed intake	High-Hg fish ≤1× observed intake§	Infeasible	–
Model 2	Drop fibre, Ca, K, P, linoleic and linolenic acids, and vitamin D	½ observed intake ≤ grams of market food groups ≤2× observed intake	High-Hg fish ≤1× observed intake§	Feasible	8·45

* Models minimising the sum of the absolute difference between the portion size of a food group in a hypothetical modelled diet and the observed diet.

† Nutrient constraints for energy, protein, carbohydrate, fat, SFA, free sugar, Ca, Cu, dietary folate, fibre, Fe, linoleic and linolenic acids, Mg, niacin, P, K, riboflavin, Na, thiamine, vitamins A, B_6_, B_12_, C and D, and Zn.

‡ See Appendix 3 for more detailed food group constraints.

§ High Hg defined as greater than 0·5 µg/g.

### Objective function

Optimisation by linear programming was used to establish an objective function defined as the ‘total departure from mean food intake’ (TDMI)^(46)^. TDMI is the sum of the absolute difference between the portion size of a food group in a hypothetical modelled diet and the observed diet^(16,25,41)^. Constraints such as meeting nutrient requirements, minimising cost and ensuring that certain foods such as TRADFOOD are either maximised or minimised were utilised in our analyses^(41,47)^. If an optimal objective function cannot be achieved, the model is considered infeasible. The program then identifies the infeasible constraints that can then be either relaxed or dropped (see Table 3).

### Energy, nutrient and cost constraints

In model 1, energy and cost were limited to being equal to or less than the observed diet or observed amounts; because this was not possible in women, the energy constraint was relaxed (i.e. increased) until a feasible model was obtained (Table 3). Constraints included a minimum of the estimated average requirements (EAR) or adequate intake for Ca, Cu, dietary folate, fibre, Fe, linoleic and linolenic acids, Mg, niacin, P, K, riboflavin, Na, thiamine, vitamins A, B_6_, B_12_, C and D, and Zn; the minimum of the acceptable macronutrient distribution range for protein, fat and carbohydrates (see Table 2); the maximum of the tolerable upper intake level (UL) for Ca, Cu, Fe, Na, P, Zn and vitamins A, B_6_, C and D (see Table 2)^(19–23,48)^. Some nutrients such as cholesterol, MUFA and PUFA and total sugar had no constraints applied. Other nutrients, i.e. SFA (≤10 %) and free sugars (≤10 %) were limited using accepted guidelines^(23,49)^. Because nutrient requirements are established by age, these were weighted by the number of participants in each age group as outlined in Table 2.

Because model 1 reduced or increased the intake of some foods to amounts unlikely to be ingested by an average individual, model 2 was designed adding constraints on food consumption. Unhealthy foods or foods that contributed little nutrient value or high fat (i.e. diet and sugar drinks, coffee whitener, salad dressings, oils, butter, margarine, fruit juice, processed meats, salt, table sauces, and savoury and sugary snacks) were limited to no less than half and no more than the amount in the observed diet whereas other foods, the maximum was no more than double the observed amount (Table 3 and Appendix 2). To attain a feasible model (i.e. model 2), we were unable to meet all the nutrient requirements, so it was necessary to remove nutrient constraints for Ca, fibre, linoleic and linolenic acids, K and vitamin D for both sexes, P in the women, Mg and vitamin A in the men (Table 3).

### Statistical analysis

All analyses were conducted using PROC OPTMODEL in SAS/STAT software version 9.4 (SAS, 2013).

## Results

### Study participants

Mean age of the participants was 46·6 (sd 15·5) years for women and 47·4 (sd 16·2) years for men (Table 4). Mean BMI was 31·2 (sd 6·59) kg/m in women and 30·4 (sd 5·51) kg/m in men, with almost 50 % of individuals categorised as obese (BMI  ≥ 30 kg/m) (Table 4). Most participants were employed (66 %), with 52 % reporting their main source of income as employment (Table 4). Household food insecurity was reported by 41 % of individuals (Table 4). Only 14 % reported eating TRADFOOD on the day of the recall (Table 4).

**Table 4.** Demographic and lifestyle characteristics of adults 19 years and older from eighteen on-reserve First Nations communities in Ontario, Canada, 2011–2012* (Numbers of participants and percentages; mean values and standard deviations)

	Women		Men	
Characteristic	*n*	%	*n*	%
*n*	856		533	
Age (years)				
Mean	46·6		47·4	
sd	15·5		16·2	
Education (years)				
Mean	11·5		10·2	
sd	3·96		3·47	
Main source of income				
Wages	443	52·1	270	51·1
Workers compensation/employment insurance†	68	8·00	54	10·2
Pension/seniors benefits	187	22·0	107	19·9
Social assistance	152	17·9	99	18·8
Employment‡	566	66·2	351	66·3
Household size (*n*)				
Mean	3·54		3·03	
sd	1·98		2·00	
BMI (kg/m)				
Mean	31·2		30·4	
sd	6·59		5·51	
BMI ≥25 kg/m	642	82·8	427	85·1
BMI ≥30 kg/m	396	51·1	240	47·8
Food insecurity§	361	43·8	192	37·6
TRADFOOD on day of 24-h recall	111	13·0	88	16·6

TRADFOOD, nutrient-dense traditional food.

* Source: First Nations Food, Nutrition and Environment Study. Data are unweighted.

† Workers’ compensation/employment insurance. Employment insurance is an insurance provided by the Canadian government for 1 year after an individual loses their job.

‡ Employment was defined as anyone in the household working for wages either part or full time.

§ Food security established using the income-related Household Food Security Survey Module adapted from food security module developed in the USA and adapted for First Nations households^(30,31)^.

### Observed diet

**Fig. 1.**
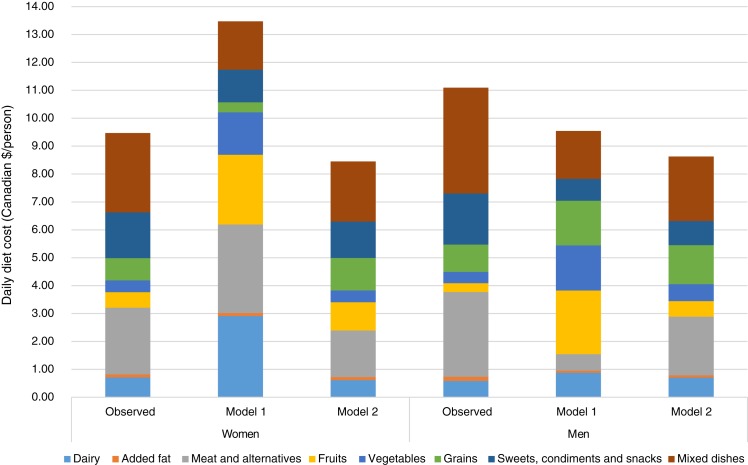
Comparison of food group costs (Canadian $/d) between the observed population diets for men and women and diets obtained through modelling.

Mean observed energy intake in women and men, respectively, was 7360 and 9422 kJ (1759 and 2252 kcal/d) (Table 2) at a total daily cost of $9·47 and $11·09 (Canadian dollars) for store-bought foods (TRADFOODS were not included in cost estimates) (Fig. 1 and Appendix 4). In women and men, the mean observed energy percenatges from macronutrients was: 47 and 45 % from carbohydrates, 37 and 38 % from fat and 17 and 18 % from protein, with fat being above the acceptable macronutrient distribution range^(23)^. Energy percentage from saturated fat was 12 % in both women and men while that from free sugar was 14 %, both higher than recommended intakes^(23,49)^. Intake of Na exceeded the UL by 20 and 54 % in women and men, respectively^(18)^. Nutrient requirements as defined by the adequate intake or the EAR were not met for fibre, vitamin D, Ca, Mg and K in both sexes, and P in women (Table 2)^(18,19,23,50)^.

The largest contribution of a food to cost and energy in the observed diet was mixed dishes for women and men (21 and 23 % of total cost and 13 % of total energy) (Appendices 3 and 5): examples of these mixed dishes included beef and vegetarian stew, pizza, lasagne, pierogis or dumplings, hamburgers and macaroni and cheese. These were followed by white bread and sweet desserts in women and beef and sweet drinks in men.

### Modelled diets

**Fig. 2.**
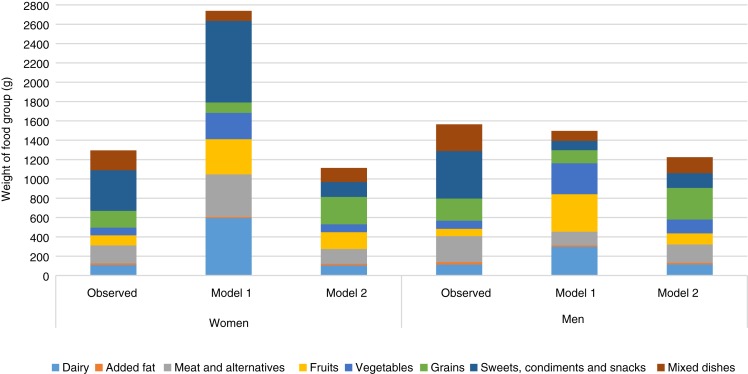
Comparison of weight of food groups between the observed population diets for women and men and diets obtained through modelling.

Model 1 in both women and men met all nutrient constraints but some of the recommended food amounts were more than twice the observed consumed quantities. In women, the modelled diet cost was higher than the observed diet (estimated as $13·47 (modelled) *v.* $9·47 (observed) in 2018) whereas it was lower for men (estimated as $9·54 (modelled) *v.* $11·09 (observed) in 2018) (Fig. 1 and Appendix 4). Similarly, energy intake was higher than the observed diet in women (8104 *v.* 7360 kJ; 1937 *v.* 1759 kcal) and lower in men (8368 *v.* 9422 kJ; 2000 *v.* 2252 kcal) (Table 2). Depending on sex, intakes of diet drinks, evaporated milk, fluid milk, fruit, meat alternatives, traditional and store-bought fish increased by more than 500 % of the observed amounts (Fig. 2). By contrast, foods such as cooking oils, butter, margarine, beef, eggs, pork, poultry and soup were reduced to small impractical amounts, not reflecting observed intake (Fig. 2).

Although model 2 was not able to meet all nutrient requirements, suggested food amounts were more reasonable. Energy intake was 7326 kJ (1751 kcal) in women (almost identical to the observed diet) and 8205 kJ (1961 kcal) in men (lower than the observed energy intake of 9422 kJ (2252 kcal)) (Table 2). Food costs were reduced from observed (women, $8·45 *v.* $9·47 and men, $8·63 *v.* $11·09) (Fig. 1 and Appendix 4). Because initially the model was infeasible, nutrient constraints were dropped for fibre, linoleic and linolenic acids, vitamin D, Ca and K in both sexes, P in women and Mg and vitamin A in men resulting in a decrease in vitamin D and Ca from observed intake in both sexes, and linoleic acid in men (all below dietary reference intakes). Despite being below recommendations, fibre and K were increased in both sexes, P in women and Mg in men^(18,19,23)^. Model 2 was able to lower Na to the UL and sugar and saturated fat to within acceptable guidelines^(18,23,49)^.

To meet most nutrient constraints, it was necessary to increase store-bought fresh or frozen fish to more than the double allowed by model 2 but the resulting amounts were within attainable ranges: for example, model 2 suggests 50 and 56 g of store-bought fresh or frozen fish per week for women and men, respectively (less than the 75 g portion recommended by the 2007 Canada's Food Guide)^(51)^. In both men and women, model 2 allowed some high-sugar items while staying within the free sugar constraint of 10 % of energy. Foods such as hot cereals, pasta and rice, potatoes and meat alternatives were doubled or almost doubled, whereas amounts of high-fat cheese, mixed dishes, soup, eggs, beef and sweet drinks were reduced (Fig. 2). Vegetable and fruit intakes were increased (Fig. 2). Na intake was reduced by the elimination of processed meats and a reduction in the amounts of soup.

## Discussion

The FNFNES is the largest Canadian study to look at the nutrition of First Nations living on reserves to date, and it provides a unique opportunity to understand current food and nutrient intakes in this population. Using food intake data (collected via 24-h recalls) from a larger sample than most other studies with First Nations in North America and optimisation modelling, we propose a diet for First Nations men and women based on a modelled dietary pattern (i.e. model 2) with realistic amounts of currently consumed foods including TRADFOOD^(52)^. Our data were collected in one season which may have influenced the TRADFOOD intake in the observed diet as TRADFOOD are harvested seasonally, and their intake is thereby influenced by the time of year.

First Nations living on-reserve have grappled with issues of TRADFOOD contamination^(53–55)^. We have taken into consideration the possible contribution of traditional fish to Hg contamination by limiting high-Hg species in our modelling, but we do not know whether the availability or palatability of these fish alternatives would influence consumption. Also, our modelling did not consider other possible contaminants, such as persistent organic pollutants, nor did it consider species availability and environmental impacts of much increased reliance on TRADFOOD in general.

The dietary patterns were modelled using EAR, adequate intake and UL as constraints whereas other investigators have used more stringent criteria such as the RDA or RDI^(56–59)^. Our decision to use the EAR was conservative but it is justified because Institute of Medicine guidelines suggest the EAR as a cut-off for groups as the RDA/RDI require meeting requirements for 97 % of a healthy population^(60)^. Despite using the lower nutrient objectives, the modelled diet was not able to meet all the nutrient requirements for this population; however, most of the nutrient intakes increased from observed amounts. Supplementation with fibre, vitamin D, Ca, Mg, K, P and linoleic acid would allow individuals to meet their dietary requirements if the modelled diet were adopted; ideally, individuals should aim to meet their nutrient needs through healthy eating patterns that include nutrient-dense foods. On the other hand, and unlike an Australian study of Indigenous individuals^(56)^, the modelled diets were able to reduce Na to the UL without increasing cost and as a result, the prevalence of high blood pressure, a risk for CVD, could be lowered^(61,62)^. Higher intakes of SFA have been associated with higher risk of CVD and diabetes, so lowering this could also help improve the health of this at-risk population^(63–65)^.

Using linear programming, our model reduced food costs by 22 % in the men and 11 % in the women, while improving or meeting nutrient requirements. This is an important consideration because First Nations individuals have lower incomes than other Canadians^(11)^ and there is a concern that a healthier diet may be prohibitively expensive^(58,66–68)^. Food costs in this study were influenced by the remoteness of communities; these were averaged over all communities sampled but would have been considerably higher in the more remote communities; for example, depending on the region, the weekly cost for a family of four ranged from $175 to $344 (Canadian dollars)^(27)^. We probably underestimated the cost of a healthy diet because the expense of harvesting TRADFOOD is a barrier to obtaining it^(69–71)^. In our study, the modelled diet did not include the cost estimates for TRADFOOD or the expense of obtaining them.

Proposing a modelled diet requires a shift away from certain commonly consumed foods (for example, processed meats where intake was reduced to zero in the present study) and a likely shift in the food system supporting the food choices, but it can reinforce TRADFOOD intake as this can be include in the constraints. Communication with health workers, dietitians and store managers in communities is important to promote the healthier alternatives with individuals while also making them more available (that is, increased physical and economic access) in community stores.

### Conclusion

A typical approach to encouraging behavioural change has been the provision of information about how and why one should choose a healthy diet; however, this has not always been successful in modifying eating behaviours because it requires the consumers to translate dietary guidelines into eating patterns^(72)^. This is a difficult task in First Nations communities where many foods are too expensive to purchase or are not available^(13)^. A more suitable approach would be to recommend a diet as similar as possible to the existing one in terms of cost and food availability. Linear programming allowed us to do this by designing a hypothetical diet that is similar in content to the existing diet^(41)^; however, we found that it may not be possible to obtain a feasible diet that meets all nutrient requirements in this Indigenous population based on the current intakes that reflect the availability/accessibility/familiarity of foods in this population. Therefore, where nutrients requirements were not met, supplementation would be required which goes against recommendations for individuals to meet their nutrient needs through healthy eating patterns that include nutrient-dense foods that are fresh or minimally processed and from meals that are prepared from scratch using these foods.

Modelling diets in this fashion allows us to suggest a modified intake using existing consumption while considering the cost of these proposed changes. This allows more practical recommendations that include important TRADFOOD. Identifying food patterns that provide needed nutrients at an affordable cost can go a long way towards mitigating social inequalities such as those that exist in First Nations peoples^(11,66)^. This, however, does not preclude the important endeavour of understanding and addressing the underlying causes of food insecurity and dietary inadequacy in First Nations peoples, foremost among them the eradication of poverty.

## Appendices

**Appendix 1.** List of foods used to calculate the cost of a nutritious food basket

Milk products 2% Milk (fresh) Cheese, processed food, cheddar, slices Cheese, mozzarella, partially skimmed, block, not slices Cheese, cheddar, block, not slices, medium Yogurt, fruit flavoured, 1 to 2% milk fat Eggs Grade A large eggs Meats, poultry and legumes Chicken legs, no back Sliced ham (11%) Beef, hip, inside round steak Beef, hip, inside (top) round roast Ground beef (lean) Canned baked beans in tomato sauce Peanuts, dry roasted Lentils, dry Peanut butter, smooth Pork chops (loin, centre cut), bone in Fish Canned flaked light tuna, packed in water Frozen fish fillets, block (sole, haddock, pollock, halibut) Canned chum or pink salmon Orange vegetables and fruit Peaches, canned halves or slices juice pack Melon or cantaloupe, raw Sweet potatoes Carrots Dark green vegetables Beans, snap, frozen Romaine lettuce Frozen mixed vegetables Broccoli Peas, green frozen Green peppers	Other vegetables and fruit Apples, Macintosh Bananas Grapes, red or green Oranges Orange juice, frozen concentrate Pears Raisins, seedless Strawberries, frozen unsweetened Canned apple juice made from concentrate, unsweetened and vitamin C added Fresh potatoes Canned kernel corn (not creamed) Turnips, yellow (rutabaga) Cabbage Cucumber Celery Iceberg lettuce Mushrooms, raw Onions, cooking Fresh tomatoes Canned whole tomatoes Vegetable juice cocktail Whole-grain products Cereal, bran flakes with raisins Oatmeal, regular quick cooking Cereal, toasted oats O's Bread, pita, whole wheat 100% Whole-wheat bread, sliced Flour, whole wheat Non-whole-grain products Social tea cookies Hot dog or hamburger buns (white) Crackers, saltine, unsalted top Enriched white bread, sliced Macaroni or spaghetti Flour, all purpose Long-grain white rice Fats and oils Rapeseed oil Salad dressing, mayonnaise-type Salad dressing, Italian Tub, margarine, non-hydrogenated

**Appendix 2.** Constraints on food amounts

Food group	Model 1	Model 2 additional proposed multiple of observed food
Diet and sweet drinks	Minimum to meet nutrient constraints	0 to 1
Coffee whitener	Minimum to meet nutrient constraints	0 to 1
High- and low-fat cheese	Minimum to meet nutrient constraints	0·5 to 2
Cream	Minimum to meet nutrient constraints	0 to 1
Canned evaporated milk	Minimum to meet nutrient constraints	0·5 to 2
High- and low-fat milk	Minimum to meet nutrient constraints	0·5 to 2
High- and low-fat yogurt	Minimum to meet nutrient constraints	0·5 to 2
Salad dressing	Minimum to meet nutrient constraints	0·5 to 1
Shortening and oil	Minimum to meet nutrient constraints	0·5 to 1
Margarine and butter	Minimum to meet nutrient constraints	0·5 to 1
Fruit juice	Minimum to meet nutrient constraints	0·5 to 1
Fruit	Minimum to meet nutrient constraints	0·5 to 2
Bannock	Minimum to meet nutrient constraints	0·5 to 2
High- and low-fibre hot cereal	Minimum to meet nutrient constraints	0·5 to 2
High- and low-fibre cereal, ready to eat	Minimum to meet nutrient constraints	0·5 to 2
Pasta and rice	Minimum to meet nutrient constraints	0·5 to 2
Potatoes	Minimum to meet nutrient constraints	0·5 to 2
Whole-grain and white bread	Minimum to meet nutrient constraints	0·5 to 2
Mixed dishes	Minimum to meet nutrient constraints	0·5 to 2
Meat alternatives	Minimum to meet nutrient constraints	0·5 to 2
Beef, eggs, fish and pork	Minimum to meet nutrient constraints	0·5 to 2
Processed meat	Minimum to meet nutrient constraints	0 to 1
Table salt and spices	Minimum to meet nutrient constraints	0·5 to 1
Table sauces	Minimum to meet nutrient constraints	0·5 to 1
Snacks	Minimum to meet nutrient constraints	0 to 1
Soup	Minimum to meet nutrient constraints	0·5 to 2
Cake, cookies and pie	Minimum to meet nutrient constraints	0 to 1
Candies	Minimum to meet nutrient constraints	0 to 1
Dairy desserts	Minimum to meet nutrient constraints	0 to 1
Sugar	Minimum to meet nutrient constraints	0·5 to 1
Non-energy sweeteners	Minimum to meet nutrient constraints	0·5 to 1
High-Hg traditional fish	Minimum to meet nutrient constraints	0·5 to 1
Low-Hg traditional fish	Minimum to meet nutrient constraints	0·5 to 2
Traditional game meat	Minimum to meet nutrient constraints	0·5 to 2
Traditional plants and fruit	Minimum to meet nutrient constraints	0·5 to 2
Vegetables, fresh and canned (not canned)	Minimum to meet nutrient constraints	0·5 to 2

**Appendix 3.** Cost and weight of foods from optimisation modelling of diet in 1387 First Nations individuals from Ontario, Canada

	Women (*n* 856)					Men (*n* 531)				
Food groups	Observed†	Model 1‡	% Observed	Model 2§	% Observed	Observed†	Model 1‡	% Observed	Model 2‖	% Observed
Cost (Canadian $)¶	9·47	13·47	142	8·45	89·2	11·09	9·54	86·0	8·63	77·8
Diet drinks (g)	76·9	836	1088*	76·9	100	61·6	61·6	100	61·6	100
Sweet drinks (g)	265·8	0·00	0·00	0·0	0·0	345	0·00	0·00	0·00	0·00
Coffee whitener (g)	1·44	0·00	0·00	1·44	100	2·60	2·60	100	2·60	100
High-fat cheese (g)	8·41	0·00	0·00*	4·21	50·0	9·06	0·00	0·00*	4·53	50·0
Low-fat cheese (g)	1·27	0·00	0·00*	1·27	100	0·09	0·09	100	0·09	100
Cream (g)	9·60	0·00	0·00	9·60	100	10·4	10·4	100	10·4	100
Canned evaporated milk (g)	4·79	74·6	1555*	4·79	100	6·05	6·05	100	12·1	200
High-fat milk (g)	6·45	115	1783*	6·45	100	7·07	7·07	100	7·07	100
Low-fat milk (g)	63·9	387	605*	63·9	100	75·3	268	356*	75·3	100
High-fat yogurt (g)	5·29	0·22	4·16*	5·29	100	0·95	0·95	100	0·95	100
Low-fat yogurt (g)	8·49	17·3	204*	8·49	100	4·91	4·91	100	4·91	100
Salad dressing (g)	3·80	11·6	306*	3·80	100	5·24	5·24	100	2·62	50·0
Shortening and oil (g)	3·15	0·00	0·00*	3·15	100	3·98	3·98	100	3·98	100
Margarine and butter (g)	8·20	0·00	0·00*	8·20	100	12·5	1·56	12·5*	6·25	50·0
Fruit juice (g)	39·0	0·00	0·00*	39·0	100	37·4	37·4	100	37·4	100
Fruit (g)	67·8	364	537*	136	200	38·3	350	914*	76·6	200
Bannock (g)	9·90	9·23	93·2	9·90	100	15·5	15·5	100	15·5	100
High-fibre hot cereal (g)	0·70	0·70	99·8	0·70	100	0·98	0·98	100	0·98	100
Low-fibre hot cereal (g)	50·2	62·8	125*	100	200	61·1	61·1	100	122	200
High-fibre cereal, ready to eat (g)	6·42	0·00	0·00*	6·42	100	6·75	6·75	100	6·75	100
Low-fibre cereal, ready to eat (g)	2·61	0·00	0·00*	2·61	100	2·44	2·44	100	2·44	100
Pasta and rice (g)	46·2	0·00	0·00*	87·4	189	69·2	0·00	0·00*	138	200
Potatoes (g)	58·7	18·8	32·0	117	200	88·1	421	478*	176	200
Whole-grain bread (g)	17·9	17·8	99·6	35·7	200	26·7	31·6	118	18·0	67·4
White bread (g)	40·0	17·8	44·4*	40·0	100	46·8	17·9	38·3*	23·4	50·0
Mixed dishes (g)	120·2	104	86·3	104	86·3	156	103	66·1	103	66·1
Meat alternatives (g)	10·4	66·4	636*	17·9	172	12·6	49·5	394*	22·7	181
Beef (g)	37·2	1·97	5·30*	21·5	57·8	57·6	1·91	3·32*	28·8	50·0
Eggs (g)	33·9	1·96	5·78*	30·9	91·2	51·2	1·92	3·75*	25·6	50·0
Fish, not canned (g)	0·06	7·14	12 600*	7·14	12 600*	1·59	8·04	505*	8·04	505*
Fish, canned (g)	1·78	119	6692*	1·78	100	1·44	1·44	100	1·44	100
Pork, not including ham (g)	11·1	1·83	16·6*	11·1	100	16·1	1·82	11·3*	8·04	50·0
Poultry (g)	44·0	2·81	6·38*	44·0	100	44·7	2·71	6·06*	22·4	50·0
Processed meat (g)	27·6	0·00	0·00	0·00	0·00	38·7	0·00	0·00	0·00	0·00
Table salt and spices (g)	6·14	0·00	0·00*	6·14	100	7·00	3·93	56·1	3·50	50·0
Table sauces (g)	4·56	0·00	0·00*	4·56	100	5·12	5·12	100	3·21	62·6
Snacks (g)	12·8	7·49	58·5*	12·8	100	15·9	15·9	100	15·9	100
Soup (g)	85·7	0·00	0·00*	42·8	50·0	121	0·00	0·00*	60·6	50·0
Cake, cookies and pie (g)	24·5	0·00	0·00	24·5	100	19·0	0·00	0·00	32·3	170
Candies (g)	9·09	0·00	0·00	9·09	100	6·38	0·86	13·5	6·38	100
Dairy desserts (g)	6·89	0·00	0·00	6·89	100	7·55	7·55	100	7·55	100
Sugar (g)	12·7	0·00	0·00*	12·7	100	22·4	0·00	0·00	22·4	100
Non-energy sweeteners (g)	0·50	0·00	0·00*	0·50	100	0·47	0·47	100	0·47	100
High-Hg traditional fish (g)	2·16	2·16	100*	2·16	100	9·65	9·65	100	9·65	100
Low-Hg traditional fish (g)	3·41	228	6686*	3·41	100	6·32	37·1	587*	6·32	100
Traditional game meat (g)	14·2	10·0	70·4	14·2	100	29·2	29·2	100	58·4	200
Traditional plants and fruit (g)	0·86	0·86	100	0·86	100	1·74	1·74	100	1·74	100
Vegetables, canned (g)	23·3	23·3	100	23·3	100	29·9	29·9	100*	36·0	120
Vegetables, fresh and frozen (g)	54·4	248	456*	56·8	54·4	52·7	288	548*	105	200

* Values are greater than double or half the observed or observed amounts for foods that would normally be eaten by individuals in this population except for those considered unhealthy such as high-sugar foods and processed meats.

† Observed is the actual or observed diet of the participants.

‡ Model 1 for both men and women was optimised using only nutrient constraints such as meeting estimated average requirements, adequate intakes, acceptable macronutrient distribution ranges except maximum 30% energy as fat, 10% energy as free sugars and 10% energy as SFA.

§ Model 2 for women included most nutrient constraints except for Ca, fibre, P and K from model 1 and additional constraints for foods limiting minimums to no less than half and maximums to double for most foods except diet and sweet drinks, coffee whitener, fats, high-sugar foods and processed meats that were limited to a maximum of the same amount as the observed.

‖ Model 2 for men included most nutrient constraints except for Ca, fibre, Mg, K and vitamins A and D from model 1 and additional constraints as for the women.

¶ Cost of foods was conservatively determined using prices from supermarkets in sampled communities for foods from Health Canada's Nutritious Food Basket (Appendix 1) and imputed from online grocery prices for other foods^(43)^.

**Appendix 4.** Comparison of cost and percentage cost for foods in observed and optimised diets in 1387 First Nations individuals from Ontario, Canada

	Women						Men					
	Observed*		Model 1†		Model 2‡		Observed*		Model 1†		Model 2§	
Food categories	Cost (Canadian $)‖	% Cost	Cost (Canadian $)‖	% Cost	Cost (Canadian $)‖	% Cost	Cost (Canadian $)‖	% Cost	Cost (Canadian $)‖	% Cost	Cost (Canadian $)‖	% Cost
Diet drinks	0·09	0·97	1·00	7·43	0·09	1·09	0·17	1·50	0·17	1·74	0·17	1·93
Sweet drinks	0·34	3·56	0·00	0·00	0·00	0·00	0·44	3·94	0·00	0·00	0·00	0·00
Coffee whitener	0·02	0·16	0·00	0·00	0·02	0·18	0·03	0·25	0·03	0·29	0·03	0·33
High-fat cheese	0·20	2·07	0·00	0·00	0·10	1·16	0·10	0·94	0·00	0·00	0·05	0·61
Low-fat cheese	0·03	0·35	0·00	0·00	0·03	0·40	0·00	0·01	0·00	0·01	0·00	0·01
Cream	0·13	1·37	0·00	0·00	0·13	1·54	0·14	1·27	0·14	1·48	0·14	1·64
Canned evaporated milk	0·11	1·14	1·68	12·5	0·11	1·28	0·14	1·23	0·14	1·43	0·14	1·58
Fluid milk	0·16	1·66	1·12	8·33	0·16	1·86	0·17	1·53	0·57	5·95	0·34	3·93
High-fat yogurt	0·03	0·34	0·00	0·01	0·03	0·38	0·01	0·05	0·01	0·06	0·01	0·06
Low-fat yogurt	0·05	0·55	0·11	0·79	0·05	0·62	0·03	0·25	0·03	0·29	0·03	0·32
Salad dressing	0·03	0·36	0·10	0·76	0·03	0·40	0·05	0·41	0·05	0·48	0·02	0·26
Shortening and oil	0·02	0·18	0·00	0·00	0·02	0·20	0·02	0·18	0·02	0·21	0·02	0·23
Margarine and butter	0·06	0·63	0·00	0·00	0·06	0·70	0·08	0·75	0·01	0·11	0·04	0·48
Fruit juice	0·09	0·90	0·00	0·00	0·09	1·01	0·08	0·68	0·08	0·79	0·08	0·87
Fruit	0·47	4·91	2·50	18·5	0·93	11·0	0·24	2·17	2·20	23·1	0·48	5·58
Bannock	0·06	0·58	0·05	0·38	0·06	0·66	0·08	0·72	0·08	0·83	0·05	0·54
High-fibre hot cereal	0·00	0·01	0·00	0·00	0·00	0·01	0·00	0·01	0·00	0·01	0·00	0·01
Low-fibre hot cereal	0·04	0·41	0·05	0·36	0·08	0·92	0·04	0·39	0·04	0·45	0·09	1·00
High-fibre cereal, ready to eat	0·08	0·88	0·00	0·00	0·08	0·99	0·08	0·72	0·08	0·84	0·16	1·86
Low-fibre cereal ready to eat	0·03	0·36	0·00	0·00	0·03	0·40	0·03	0·26	0·03	0·30	0·03	0·34
Pasta and rice	0·08	0·79	0·00	0·00	0·14	1·68	0·11	1·01	0·00	0·00	0·22	2·61
Potatoes	0·17	1·77	0·05	0·39	0·33	3·92	0·23	2·09	1·11	11·61	0·46	5·37
Whole-grain bread	0·10	1·03	0·10	0·72	0·20	2·31	0·13	1·21	0·16	1·66	0·25	2·94
White bread	0·25	2·61	0·11	0·81	0·25	2·92	0·27	2·40	0·10	1·07	0·13	1·54
Mixed dishes	2·01	21·21	1·73	12·9	1·73	20·5	2·60	23·4	1·72	18·0	1·72	19·9
Meat alternatives	0·06	0·62	0·37	2·76	0·10	1·19	0·06	0·58	0·25	2·67	0·13	1·50
Beef	0·66	6·98	0·04	0·26	0·38	4·53	0·96	8·68	0·03	0·33	0·91	10·57
Eggs	0·26	2·79	0·02	0·11	0·24	2·86	0·37	3·30	0·01	0·14	0·18	2·12
Fish, not canned	0·00	0·02	0·20	1·49	0·20	2·38	0·04	0·37	0·21	2·18	0·70	8·10
Fish, canned	0·04	0·39	2·49	18·5	0·04	0·44	0·03	0·25	0·03	0·29	0·03	0·32
Pork, not including ham	0·18	1·91	0·03	0·22	0·18	2·14	0·24	2·17	0·03	0·29	0·12	1·39
Poultry	0·53	5·55	0·03	0·25	0·53	6·22	0·49	4·42	0·03	0·31	0·03	0·34
Processed meat	0·66	6·97	0·00	0·00	0·00	0·00	0·85	7·62	0·00	0·00	0·00	0·00
Table salt and spices	0·17	1·81	0·00	0·00	0·17	2·06	0·19	1·76	0·11	1·14	0·10	1·13
Table sauces	0·03	0·35	0·00	0·00	0·03	0·39	0·04	0·34	0·04	0·40	0·02	0·22
Snacks	0·28	2·98	0·17	1·23	0·28	3·34	0·35	3·14	0·35	3·65	0·17	2·02
Soup	0·84	8·90	0·00	0·00	0·42	4·98	1·19	10·7	0·00	0·00	0·60	6·91
Cake, cookies, pies and candies	0·58	6·17	0·00	0·00	0·58	6·91	0·45	4·10	0·02	0·23	0·21	2·46
Dairy desserts	0·06	0·60	0·00	0·00	0·06	0·67	0·06	0·56	0·06	0·65	0·06	0·72
Sugar	0·05	0·56	0·00	0·00	0·05	0·63	0·09	0·84	0·00	0·00	0·09	1·08
Non-energy sweeteners	0·01	0·13	0·00	0·00	0·01	0·14	0·01	0·10	0·01	0·12	0·01	0·13
High-Hg traditional fish	0·00	0·00	0·00	0·00	0·00	0·00	0·00	0·00	0·00	0·00	0·00	0·00
Low-Hg traditional fish	0·00	0·02	0·00	0·00	0·00	0·00	0·00	0·00	0·00	0·00	0·00	0·00
Traditional game meat	0·00	0·00	0·00	0·00	0·00	0·00	0·00	0·00	0·00	0·00	0·00	0·00
Traditional plants and fruit	0·00	0·00	0·00	0·00	0·00	0·00	0·00	0·00	0·00	0·00	0·00	0·00
Vegetables, canned	0·11	1·17	0·11	0·82	0·11	1·31	0·13	1·22	0·13	1·41	0·07	0·78
Vegetables, fresh and frozen	0·31	3·28	1·41	10·5	0·31	3·66	0·27	2·44	1·48	15·5	0·54	6·27

* Observed is the actual or observed diet of the participants.

† Model 1 for both men and women was optimised using only nutrient constraints such as meeting estimated average requirements, adequate intakes, acceptable macronutrient distribution ranges except maximum 30% energy as fat, 10% energy as free sugars and 10% energy as SFA.

‡ Model 2 for women included most nutrient constraints except for Ca, fibre, P and K from model 1 and additional constraints for foods limiting minimums to no less than half and maximums to double for most foods except diet and sweet drinks, coffee whitener, fats, high-sugar foods and processed meats that were limited to a maximum of the same amount as the observed.

§ Model 2 for men included most nutrient constraints except for Ca, fibre, Mg, K and vitamins A and D from model 1 and additional constraints as for the women.

‖ Cost was conservatively determined using prices from supermarkets in sampled communities for foods from Health Canada's Nutritious Food Basket (Appendix 1) and imputed from online grocery prices for other foods^(43)^.

**Appendix 5.** Comparison of energy and energy percentage for foods in observed and optimised diets in 1387 First Nations individuals from Ontario, Canada

	Women						Men					
	Observed*		Model 1†		Model 2‡		Observed*		Model 1†		Model 2§	
Food categories	Energy (kcal)‖	% Energy	Energy (kcal)‖	% Energy	Energy (kcal)‖	% Energy	Energy (kcal)‖	% energy	Energy (kcal)‖	% Energy	Energy (kcal)‖	% Energy
Diet drinks	1·38	0·08	15·0	0·77	1·38	0·08	1·61	0·07	1·61	0·08	1·61	0·08
Sweet drinks	105	6·00	0·00	0·00	0·00	0·00	137	6·06	0·0	0·00	0·00	0·00
Coffee whitener	7·68	0·44	0·00	0·00	7·68	0·44	13·3	0·58	13·3	0·66	13·3	0·67
High-fat cheese	30·5	1·75	0·00	0·00	15·3	0·87	33·4	1·47	0·00	0·00	16·7	0·85
Low-fat cheese	1·73	0·1	0·00	0·00	1·73	0·10	0·27	0·01	0·27	0·01	0·27	0·01
Cream	14·6	0·83	0·00	0·00	14·6	0·83	16·1	0·71	16·1	0·80	16·1	0·82
Canned evaporated milk	4·79	0·27	74·50	3·82	4·79	0·27	5·73	0·25	5·73	0·29	11..5	0·58
Fluid milk	35·6	2·03	269	13·8	35·6	2·03	41·9	1·84	137	6·82	41·9	2·13
High-fat yogurt	5·55	0·32	0·23	0·01	5·55	0·32	1·18	0·05	1·18	0·06	1·18	0·06
Low-fat yoghurt	7·14	0·41	14·6	0·75	7·14	0·41	3·99	0·18	3·99	0·20	3·99	0·20
Salad dressing	17·1	0·98	52·1	2·68	17·1	0·98	25·9	1·14	25·9	1·29	12·9	0·66
Shortening and oil	27·8	1·59	0·00	0·00	27·8	1·59	35·3	1·55	35·3	1·75	35·3	1·79
Margarine and butter	53·2	3·04	0·00	0·00	53·2	3·04	87·5	3·85	10·9	0·54	43·8	2·22
Fruit juice	18·3	1·04	0·00	0·00	18·3	1·05	17·6	0·78	17·6	0·88	17·6	0·89
Fruit	37·5	2·14	201	10·3	74·9	4·28	24·5	1·08	224	11·13	48·9	2·48
Bannock	26·8	1·53	25·0	1·28	26·8	1·53	42·5	1·87	42·5	2·12	42·5	2·15
High-fibre hot cereal	2·69	0·15	2·69	0·14	2·69	0·15	3·66	0·16	3·66	0·18	3·66	0·19
Low-fibre hot cereal	41·9	2·39	52·4	2·69	83·8	4·79	49·0	2·16	49·0	2·44	98·0	4·97
High-fibre cereal, ready to eat	24·2	1·38	0·00	0·00	24·2	1·38	26·4	1·16	26·4	1·31	26·4	1·34
Low-fibre cereal, ready to eat	10·2	0·58	0·00	0·00	10·2	0·58	9·50	0·42	9·50	0·47	9·50	0·48
Pasta and rice	69·7	3·98	0·00	0·00	132	7·54	102	4·50	0·00	0·00	204	10·3
Potatoes	78·1	4·47	25·0	1·28	156	8·93	110	4·83	524	26·1	219	11·1
Whole-grain bread	50·2	2·87	50·0	2·57	100	5·74	74·2	3·27	87·9	4·37	50·0	2·53
White bread	112	6·43	50·0	2·57	113	6·43	131	5·75	50·0	2·49	65·3	3·31
Mixed dishes	232	13·2	200	10·3	200	11·4	303	13·3	200	9·95	200	10·1
Meat alternatives	29·1	1·66	185	9·52	50·0	2·86	27·7	1·22	109	5·43	50·0	2·53
Beef	94·3	5·39	5·00	0·26	54·5	3·12	151	6·66	5·00	0·25	75·6	3·83
Eggs	51·9	2·97	3·00	0·15	47·4	2·71	79·9	3·52	3·00	0·15	39·9	2·02
Fish (not canned)	0·08	0	10·0	0·51	10·0	0·57	1·98	0·09	10·0	0·50	10·0	0·51
Fish, canned	2·43	0·14	162	8·34	2·43	0·14	2·18	0·10	2·18	0·11	2·18	0·11
Pork (not including ham)	30·2	1·72	5·00	0·26	30·2	1·72	44·1	1·95	5·00	0·25	22·1	1·12
Poultry	94·1	5·37	6·00	0·31	94·1	5·37	99·1	4·37	6·00	0·30	49·6	2·51
Processed meat	82·6	4·72	0·00	0·00	0·00	0·00	120	5·27	0·00	0·00	0·00	0·00
Table salt and spices	4·20	0·24	0·00	0·00	4·20	0·24	4·60	0·20	2·58	0·13	2·30	0·12
Table sauces	4·04	0·23	0·00	0·00	4·04	0·23	5·07	0·22	5·07	0·25	3·17	0·16
Snacks	64·6	3·69	37·8	1·94	64·6	3·69	80·8	3·56	80·8	4·02	80·8	4·09
Soup	42·5	2·43	0·00	0·00	21·2	1·21	59·4	2·62	0·00	0·00	29·7	1·50
Cake, cookies, pies and candies	109	6·25	0·00	0·00	109	6·25	92·9	4·09	3·33	0·17	141	7·14
Dairy desserts	12·4	0·71	0·00	0·00	12·4	0·71	15·0	0·66	15·0	0·75	15·0	0·76
Sugar	46·3	2·64	0·00	0·00	46·3	2·64	81·4	3·58	0·00	0·00	81·4	4·12
Non-energy sweeteners	1·64	0·09	0·00	0·00	1·64	0·09	1·54	0·07	1·54	0·08	1·54	0·08
High-Hg traditional fish	3·08	0·18	3·08	0·16	3·08	0·18	13·5	0·59	13·5	0·67	13·5	0·68
Low-Hg traditional fish	5·54	0·32	371	19·0	5·54	0·32	9·91	0·44	68·2	3·39	19·9	1·01
Traditional game meat	21·8	1·24	15·3	0·79	21·8	1·24	41·4	1·83	41·4	2·06	82·9	4·20
Traditional plants and fruit	0·5	0·03	0·50	0·03	0·50	0·03	1·54	0·07	1·54	0·08	1·54	0·08
Vegetables, canned	11·3	0·64	11·3	0·58	11·3	0·64	15·3	0·67	15·3	0·76	18·4	0·93
Vegetables, fresh and frozen	22·1	1·26	101	5·17	22·1	1·26	24·8	1·09	136	6·75	49·5	2·51

* Observed is the actual or observed diet of the participants.

† Model 1 for both men and women was optimised using only nutrient constraints such as meeting estimated average requirements, adequate intakes, acceptable macronutrient distribution ranges except maximum 30% energy as fat, 10% energy as free sugars and 10% energy as SFA.

‡ Model 2 for women included most nutrient constraints except for Ca, fibre, P and K from model 1 and additional constraints for foods limiting minimums to no less than half and maximums to double for most foods except diet and sweet drinks, coffee whitener, fats, high-sugar foods and processed meats that were limited to a maximum of the same amount as the observed.

§ Model 2 for men included most nutrient constraints except for Ca, fibre, Mg, K and vitamins A and D from model 1 and additional constraints as for the women.

‖ To convert kcal to kJ, multiply the kcal value by 4·184.
